# Enhancing Treatment Outcomes in Atrial Fibrillation: A Contemporary Literature Review of the Impact of Optimizing Patient Well-Being in Treatment Management

**DOI:** 10.7759/cureus.62321

**Published:** 2024-06-13

**Authors:** Mukosolu F Obi, Manjari Sharma, Vikhyath Namireddy, Vyoma Patel, Arianna Reinberg Palmar, Ngozi T Kanu

**Affiliations:** 1 Internal Medicine, Wyckoff Heights Medical Center, Brooklyn, USA; 2 Medicine, St. George's University School of Medicine, True Blue, GRD

**Keywords:** obesity and diabetes, obstructive sleep apnoea, standard of care, comorbidities for atrial fibrillation, atrial fibrillation management, atrial fibrillation (af), atrial arrhythmia

## Abstract

Atrial fibrillation (AF) is the most common heart rhythm disorder, defined by an irregular and rapid heartbeat. It is the most prevalent cardiac arrhythmia in the United States, characterized by irregular heartbeats due to asynchrony between atrial and ventricular contractions. AF can be categorized as paroxysmal or persistent and, as such, poses significant health risks, including heart failure and stroke. Factors like age, sex, lifestyle, and existing health conditions elevate AF risk. There have been a lot of debates around AF risk management and its impact on prognosis. This literature review aims to explore the influence of addressing modifiable risk factors in AF patients on its morbidity and mortality, exploring various treatment options and their effectiveness. Current guidelines suggest rate control and anticoagulation for persistent AF with medications like beta blockers and non-vitamin K oral anticoagulants. Catheter ablation for rhythm control is contentious. Studies on supplemental treatments, lifestyle changes, and managing comorbidities show mixed results, necessitating further research for comprehensive treatment effectiveness in AF patients, which this literature review will discuss.

## Introduction and background

The prevalence and incidence of atrial fibrillation (AF) have been increasing in the United States and globally, even with undiagnosed AF. An estimated and reported increase was noted using the back-calculation methodology. The estimated global prevalence was 50 million in 2020; in the United States, it was 5.2 million in 2010, and it is expected to rise to 12.1 million in 2030 [1]. AF is associated with a 1.5- to two-fold increased risk of death. AF is the most commonly reported arrhythmia, predisposing patients to an increased risk of stroke and heart failure. Paroxysmal and persistent are the two subtypes of AF. Paroxysmal AF is defined as AF that terminates spontaneously within seven days of onset, regardless of whether it is treated or not. This definition helps clinicians categorize AF and determine appropriate management strategies based on the pattern and frequency of episodes. Persistent AF is an AF that sustains beyond seven days and requires medical intervention or cardioversion (pharmacological or electrical) to restore normal sinus rhythm. Long-standing persistent AF is defined as AF that has persisted for more than or equal to 12 months, meaning that efforts to restore normal sinus rhythm have not succeeded [2]. Patients with long-standing AF often have structural changes in the atria and may have a higher burden of symptoms and a higher risk of complications compared to those with shorter durations of AF.

The primary treatment goals for AF are rate, rhythm control, and anticoagulation (AC). However, considering modifiable risk factors alongside other therapeutic strategies has shown significant efficacy in treating AF. Modifiable risk factors include smoking cessation, sleep studies, unhealthy alcohol consumption, weight loss with a BMI >27 kg/m2, etc. [1]. Rate control, achieved through medications like beta blockers and calcium channel blockers, aims to bring the heart rate to a normal range (60-100 beats per minute), alleviating symptoms caused by the rapid, irregular heart rhythm in AF. Anticoagulants, such as non-vitamin K oral anticoagulants (NOAC) or vitamin K antagonists (VKA), serve as preventive measures against stroke, a complication associated with AF [3,4]. Risk stratification for thromboembolic events using validated clinic risk scores such as CHA2DS2-VASc (which indicates congestive heart failure, hypertension, age ≥75 years (doubled), diabetes mellitus, prior stroke or transient ischemic attack or thromboembolism (doubled), vascular disease, age 65 to 74 years, sex category) has been effective and beneficial in treatment strategy. Other valid calculators are the Anticoagulation and Risk Factors in Atrial Fibrillation and Global Anticoagulant Registry in the Field Atrial Fibrillation risk scores. AC is considered in men, with scores 1 and 2 for women [1]. AC increases the risk of bleeding, and given that most people with AF are elderly or have previous bleeding risk, it is important to address the risks and benefits of AC and comply with informed medical decisions [1]. Using direct oral anticoagulants appears to provide a more advantageous overall outcome in preventing thromboembolic events and reducing the likelihood of major bleeding when compared to using VKAs [1].

For patients who have an absolute contraindication for AC, implanting a WATCHMAN device (Boston Scientific Corporation, Massachusetts, USA) for transcatheter left atrial appendage occlusion demonstrated a minimal incidence of stroke after one year, particularly in elderly individuals with non-valvular AF who were at a heightened risk for stroke or bleeding based on their medical background. This innovative device obstructs a small section of the heart, thereby mitigating the risk of hazardous clot formation, specifically in non-valvular AF, thus serving as a preventive measure against left atrial thromboembolism [5].

This review aims to examine how, while the current conventional approach to treating AF revolves around managing heart rate and rhythm and administering anticoagulants, incorporating the identification and treatment of modifiable risk factors into AF management can significantly enhance outcomes and diminish AF-related complications. Neglecting to address these factors is closely linked to a heightened recurrence and incidence of AF, underscoring the importance of their integration into treatment strategies.

## Review

This comprehensive literature review emphasizes the significance of addressing modifiable risk factors such as sleep apnea, alcohol use, obesity, and a sedentary lifestyle in the effective management of AF. By integrating the treatment of these factors alongside standard therapies like AC and rate/rhythm control, the goal is to minimize hospitalizations and associated risks.

The review's inclusion criteria encompassed studies that looked at the impact of obesity, alcoholism, physical inactivity, and sleep apnea treatments in conjunction with AF management to alleviate symptoms, reduce recurrence, and prevent progression to persistent AF. The review used observational and case-crossover studies with criteria such as BMI >27 kg/m2, ≤3 standard drinks weekly, and at least 210 minutes of exercise per week to demonstrate the benefits of addressing these risk factors in mitigating AF burden. A search across PubMed and Google Scholar yielded numerous relevant articles, with a total of 22 literature studies reviewed and summarized in Table 1.

**Table 1:** Results of the literature review conducted AHA: American Heart Association, ACC: American College of Cardiology, AF: atrial fibrillation, NOACs: non-vitamin K oral anticoagulants, VKA: vitamin K antagonists, OSA: obstructive sleep apnea, AC: anticoagulation

Authorship and year of publication	Objective of study	Type of treatment utilized	Conclusion
Joglar et al. (2023) [1]	To summarize the AHA/ACC guidelines for the management of patients with AF	N/A	N/A
Markides and Schilling (2003) [2]	To discuss the pathophysiology of AF and its pharmacological treatment	N/A	Pharmacological therapy remains the mainstay of treatment for the majority of patients. The optimum treatment strategy for patients with persistent AF remains controversial, with some clinicians favoring rhythm control and others rate control. AC or antiplatelet therapy for stroke prevention forms an integral part of the treatment of patients with AF and risk factors for thromboembolism
Lloyd-Jones 2004) [3]	To provide information about the epidemiology and treatment options of AF	N/A	N/A
Bassand et al. (2021) [4]	To indicate that there is a lower risk of bleeding and death in patients taking NOACs compared to VKA	AC	The incidence of any bleeding and all-cause mortality was lower with NOACs compared to VKAs
Magdi et al. (2021) [5]	To show that the WATCHMAN device can be used as an alternative in patients who are in lifelong AC due to absolute contraindication	Watchman device	Watchman implantation reduces the risk of bleeding in patients with absolute contraindications to AC. Although a short course of VKA followed by antiplatelet is required or antiplatelet alone after implantation of the device
Gami et al. (2004) [6]	To show that OSA is associated with an increased risk of AF	Lifestyle modification and continuous positive airway pressure	There is a strong association between OSA and AF, with increased AF burden if left untreated. Since OSA is sometimes undiagnosed, patients with AF should be evaluated for OSA especially if they have components of obesity
Jennings et al. (2013) [7]	To indicate that cardiac myocyte remodeling with downregulation of connexin proteins predisposes to AF	Lifestyle modification is a view of risk factors that predispose to connexin downregulation. Also, medication targeting connexin like rotigaptide	Alteration in connexin physiology has been associated with sustained AF
Linz et al. (2018) [8]	To show the connection between OSA and AF	Lifestyle modification	Recognizing that OSA is a substrate for AF, increases the effectiveness of AF treatment. As the presence of OSA decreases the efficacy of catheter-based and pharmacological treatment of AF
Alpert et al. (2016) [9]	To indicate that obesity can alter cardiac morphology thereby increasing the risk of AF	Lifestyle modification	Excessive adipose tissue can alter cardiac morphology predisposing to cardiovascular disease and weight loss can reverse this effect
Lavie et al. [10]	To affirm that obesity increases the risk of AF	Lifestyle modification	AF and obesity are interrelated. Although some studies expressed the existence of an obesity paradox, weight loss, and increased physical activity can reduce the recurrence of AF
Chung et al. (2020) [11]	To review associated risk factors of AF and ways to combat it, to alleviate the complications of AF	Lifestyle modification	Lifestyle habits that increase the risk of AF should be considered as chronic disease and require intervention and encouragement of maintenance to produce a long-term benefit in the treatment of AF
Jiang et al. (2022) [12]	To explore the association between alcohol consumption and AF in different genders	Lifestyle modification	The dose-dependent metanalysis study concluded that there is a difference in the risk of AF and alcohol consumption in different genders. With linear association in men compared to women
Voskoboinik et al. (2016) [13]	To demonstrate the link between alcohol consumption and AF	Lifestyle modification	Alcohol consumption predisposes to AF through direct effects of autonomic dysregulation and cellular remodeling

Discussion

The bedrock of AF treatment revolves around evaluating and addressing stroke risk factors, enhancing the management of all modifiable risk elements, and effectively managing AF symptoms. A vital component of care involves tackling various medical conditions and situations that elevate the probability of a specific outcome while motivating individuals to alter their behavior. This strategy aims to diminish the risk of developing AF and mitigate its adverse impacts.

Pathophysiology

AF often originates from electrical impulses/ectopic foci originating from the pulmonary veins of the left atrium or re-entrant electrical pathways/circuits due to fibrosis or scarring in the atrial tissues, disrupting the normal conducting pathways. Structural and electrical remodeling can be prevented if lifestyle changes are implemented.

Sleep-disordered breathing (SDB): SDB, such as obstructive sleep apnea (OSA), increases the risk of AF. Based on observational studies, the prevalence of OSA is higher in patients with AF compared to the general population, as it has been discovered to lead to arrhythmogenesis and impair treatment efficacy [6]. In patients with OSA acute intermittent episodes of apnea-related atrial electrophysiological changes due to intermittent hypoxemia, hypercapnia during obstructive breathing effort can increase fluctuation in intrathoracic pressure, with exciting chemoreceptors and sympathovagal activation predisposing to AF. The nocturnal activation of the sympathetic system persists during wakefulness in OSA, often leading to AF [7,8]. These factors collectively stimulate AF triggers and create a complex, dynamic substrate for AF during sleep. Over time, repeated episodes of OSA lead to structural remodeling and alterations in atrial electrical conduction.

Furthermore, increased breathing efforts against an obstructed airway during apnea increase transmural pressures in cardiac chambers, triggering stretch-activated atrial ion channels and promoting AF [7]. Gap junctions are specialized intercellular connections that facilitate direct communication between adjacent cells. Connexin is a gap junction protein found in cardiac myocytes and helps in cardiac conduction. The downregulation of connexin reduces the number of gap junctions, leading to slower and more uneven atrial conduction velocity and repolarization. This then creates regional functional conduction blocks that can cause re-entry and promote AF [7]. Some observational studies indicated that OSA reduces the effectiveness of catheter-based and pharmacological antiarrhythmic treatments. Also, nonrandomized studies show that treating OSA with continuous positive airway pressure can help maintain sinus rhythm after electrical cardioversion and catheter ablation in AF patients. Given the high prevalence of OSA in AF patients, often without reported daytime sleepiness, sleep studies should be considered for those considering rhythm control strategies [6].

Obesity and physical inactivity: There is an increase in the prevalence of cardiovascular disease, given the increased incidence of obesity and AF. Obesity increases the risk of AF as a result of electro-anatomical remodeling [10,11]. Obesity directly causes changes in the atrial myocardium, forming the AF substrate. Epicardial adipose tissue (EAT) and pericardial fat, which is brown fat, have been implicated in the development of AF. These fats are biologically active and secrete cytokines with remodeling factors such as activating A, follistatin, transforming growth factors 1-3, matrix metalloproteinases 1-13, and adipocytokines like adiponectin, leptin, resistin, visfatin, and omentin [10]. Pericardial fat and EAT are found more predominately around pulmonary veins as they enter the left atrium. EATs also have a dense network of autonomic ganglia with parasympathetic nerve fiber predominance [10]. Multiple studies have shown that EAT and pericardial fat can predispose to paroxysmal and persistent AF, even with a high recurrence rate of AF after catheter ablation. The exact mechanisms through which increased pericardial fat and EAT contribute to the development of AF remain unclear. However, it is suggested that the enhanced sympathetic or parasympathetic activity associated with the dense innervation of fat deposits adjacent to the left atrium and pulmonary veins may play a significant role [10]. Increased adipose tissue can lead to a hypoxic condition due to insufficient capillary supply. This hypoxia triggers inflammation and the release of cytokines, which can alter adipokine levels, disrupt ion channel function, and impair calcium homeostasis [9]. These changes contribute to atrial fibrosis and promote arrhythmias in the pulmonary veins, ultimately increasing the risk of AF [10]. Given these findings, it is imperative to implement weight loss with the target of a normal BMI in patients with AF. Several observational studies have demonstrated a decrease in AF burden with weight loss. Multiple trials, for example, the Long-Term Effect of Goal-Directed Weight Management in an Atrial Fibrillation Cohort Trial, demonstrated that patients who underwent goal-directed weight loss and risk factor management over four years were analyzed based on the percentage of body weight lost. It was found that those who lost and maintained at least 10% of their body weight were six times more likely to remain free of arrhythmia than those who lost less than 3% or gained weight. Also, weight fluctuations exceeding 5% were independently associated with AF recurrence, diminishing weight loss benefits [11]. However, the obesity paradox is well known, in which patients who are overweight and obese have a better prognosis than leaner patients with the same degree of AF burden [10]. Physical activity and weight loss are still beneficial for patients with AF. Recent evidence shows that weight loss and physical activity programs reduce AF recurrences in patients with a history of the condition. Additionally, higher levels of cardiorespiratory fitness are linked to the primary prevention of AF [11].

Alcohol consumption: Based on a meta-analysis, the average consumption of one to two alcoholic drinks increases the risk of AF [12]. This study went further to indicate a linear association between alcohol consumption and AF in men compared to women. Most alcoholic emergencies precipitate AF in 35% to 62% of cases [13]. Alcohol consumption is categorized as follows: light (less than seven standard drinks per week), moderate (seven to 21 standard drinks per week), and heavy (more than 21 standard drinks per week), with one standard drink containing roughly 12 grams of alcohol [13]. Alcohol can cause immediate electrophysiological changes, including shortening of the atrial refractory period and increased atrial conduction velocity, which can promote the initiation of AF. Long-term alcohol consumption can lead to alterations in ion channel function and calcium handling, contributing to the maintenance of AF [13]. Studies have shown that alcohol consumption has both a parasympathetic and sympathetic effect on cardiac myocytes, leading to AF. Habitual drinkers, known as light-moderate drinkers, have parasympathetic autonomic modulation in which vagal stimulation shortens atrial refractoriness, which can then result in excitatory or fibrillatory conductions [13]. Given alcohol-induced sympathetic activation, this process causes the spontaneous release of intracellular calcium from the sarcoplasmic reticulum, promoting arrhythmogenesis.

Cellular effect of alcohol: Alcohol and its metabolite acetaldehyde can cause cardiotoxicity, leading to cardiac fibrosis and affecting the atrial excitation-contraction coupling pathway [13]. Habitual drinking and binge drinking both increase the risk of developing AF and its recurrence in those who continue to consume alcohol. While small amounts of alcohol may offer some cardioprotective benefits, these do not apply to AF.

The majority of AF patients typically have additional health issues or lifestyle choices that increase their chance of developing AF. Combining lifestyle modifications with other AF treatments has a synergistic effect that can significantly improve AF management, prevention, and burden. In addition to treating the underlying causes of AF, a complete strategy incorporating regular exercise, weight loss, and efficient treatment of OSA enhances cardiovascular health. As AF management aims to relieve symptoms and avoid cardiovascular complications, a synergistic strategy combining rate/rhythm control, catheter ablation, and lifestyle modification can significantly minimize AF recurrence and burden. This all-encompassing approach can lessen the frequency, length, and intensity of AF episodes. Modifications to one's lifestyle immediately address the pathophysiological processes that underlie AF. Patients can obtain significant decreases in the burden and recurrence of AF by targeting these pathways. Prioritizing these interventions in treatment plans can improve the long-term results for individuals with AF, highlighting lifestyle's vital role in preserving cardiovascular health. Ultimately, switching to a healthy lifestyle is a very successful strategy for treating AF. Losing weight, exercising regularly, and controlling OSA can all significantly impact and highlight how crucial lifestyle changes are to reaching and maintaining improved cardiovascular health.

## Conclusions

AF is a chronic condition linked to increased morbidity and mortality, including stroke, heart failure, myocardial infarction, and all-cause mortality. Investigating optimal AF management is crucial for improving the prognosis of individuals with the condition and those at high risk. Lifestyle modifications can control or eliminate many contributing risk factors, as suggested by various studies. However, most AF patients have multiple coexisting risk factors influencing their health. Further research using a multivariable and integrated approach is necessary to understand how simultaneous therapies targeting numerous risk factors affect AF incidence and overall patient well-being. Nevertheless, current literature emphasizes the importance of controlling highly associated risk factors to ensure the overall well-being of AF patients.
